# The influence of anterior cervical discectomy and fusion surgery on cervical muscles and the correlation between related muscle changes and surgical efficacy

**DOI:** 10.1186/s13018-024-04605-2

**Published:** 2024-03-16

**Authors:** Chong Sun, Hongfei Xiang, Xiaolin Wu, Bohua Chen, Zhu Guo

**Affiliations:** 1 Department of Spinal Surgery, The Affiliated Hospital of Qingdao University, Qingdao, Shandong China; 2 Department of Trauma Surgery, The Affiliated Hospital of Qingdao University, Qingdao, Shandong China

**Keywords:** Anterior cervical discectomy and fusion, Cervical longus, Cervical extensor muscles, Clinical efficacy

## Abstract

**Background:**

Anterior cervical discectomy and fusion surgery (ACDF) is a common technique in treating degenerative cervical spondylosis. This study is to evaluate the changes of cervical muscles after ACDF and analyze the correlation between related muscle changes and clinical efficacy.

**Methods:**

Sixty-five postoperative patients (single-level ACDF) with cervical spondylotic myelopathy from January 2013 to December 2022 were analyzed. The measured parameters include: the axial section of longus colli cross-sectional area (AxCSA), the volume of cervical longus, the ratio of long and short diameter line (RLS), the cervical extensor cross-sectional area (CESA), the vertebral body area (VBA), and the CESA/VBA. The visual analog scale (VAS), modified Japanese Orthopedic Association score (mJOA), and neck disability index (NDI) were evaluated. The changes in muscle morphology were analyzed, and the correlation analysis was conducted between morphological changes and function scores.

**Results:**

The postoperative AxCSA of surgical segment (3rd month, 12th month, and the last follow-up) was decreased compared to preoperative (141.62 ± 19.78), and the differences were significant (*P* < 0.05). The corresponding data reduced to (119.42 ± 20.08) mm^2^, (117.59 ± 19.69) mm^2^, and (117.41 ± 19.19) mm^2^, respectively (*P* < 0.05). The RLS increased, and the volume of cervical longus decreased significantly after surgery (*P* < 0.05). Negative correlation was found between postoperative volume of cervical longus and VAS at the 3rd month (*r* = − 0.412), 12th month (*r* = − 0.272), and last follow-up (*r* = − 0.391) (*P* < 0.05). Negative correlation existed between postoperative volume of cervical longus and NDI at the 3rd month (*r* = − 0.552), 12th month (*r* = − 0.293), and last follow-up (*r* = − 0.459) (*P* < 0.05).

**Conclusion:**

The volume of cervical longus decreased and its morphology changed after ACDF surgery. The mainly affected muscle was the cervical longus closing to the surgical segment. Negative correlation was found between the postoperative volume of cervical longus and function scores (VAS and NDI).

## Introduction

Cervical spondylotic myelopathy (CSM) stands as the prevailing manifestation of spinal cord dysfunction in the adult population. This malady encompasses an array of clinical manifestations arising from the stimulation and compression of the spinal cord, emanating from the degeneration of cervical intervertebral disks or pathological alterations within the cervical bone structures [1, 2]. In instances where a patient afflicted with single-segment CSM manifests severe and unrelieved symptoms and signs despite conservative interventions, clinical practice typically involves resorting to surgical modalities, such as anterior cervical discectomy and fusion (ACDF) or artificial cervical disk replacement (ACDR). Within the array of surgical procedures employing the anterior cervical approach, ACDF has emerged as a prominent technique for addressing degenerative cervical spondylosis. This preference is attributed to its demonstrably superior surgical efficacy and heightened safety profile. The objective of this surgical intervention is to comprehensively decompress and efficiently fuse the afflicted cervical vertebra segments. This approach aims to alleviate nerve compression effectively, reinstate the intervertebral space’s dimensions, rectify the sagittal balance of the cervical spine, and ultimately ameliorate the clinical symptoms experienced by the patient [3, 4].

The equilibrium and stability of the cervical spine are influenced by both the cervical intervertebral disk and cervical ligaments. Simultaneously, the cervical paravertebral muscles assume a pivotal role in upholding the stability of the cervical spine [5]. In recent years, numerous pertinent studies have undertaken substantial investigations into the role of pericervical muscles in the normal biomechanics of the cervical spine and the preservation of cervical stability [6, 7]. Nevertheless, to date, there is a paucity of the literature addressing the alterations in pericervical muscles following cervical spine surgery. Specifically, scant attention has been given to the impact of ACDF surgery on cervical longus and cervical extensor muscles, and the correlation between changes in cervical paravertebral muscles and postoperative therapeutic outcomes remains unexplored. Previous investigations have underscored the substantial role played by deep neck flexors, such as cervical longus and longus capitis, as well as cervical extensor muscles in sustaining cervical spine stability. Moreover, it has been established that patients with chronic neck pain often exhibit dysfunction in these deep neck flexors. Concurrently, the injury incurred by the posterior cervical muscles due to posterior cervical surgery can give rise to postoperative neck pain [8, 9]. While ACDF surgery mitigates the risk of direct injuries to posterior cervical muscles, the diminished mobility arising from the fusion of cervical segments may potentially impact the posterior extensor muscles of the cervical spine. Consequently, alterations in cervical paravertebral muscles following ACDF surgery could be considered potential contributors to the onset of chronic neck pain and axial discomfort subsequent to cervical surgery [10].

## Patients and methods

This retrospective study systematically examined the clinical and imaging data of patients diagnosed with single-segment CSM who underwent ACDF surgery within our department. The dual objectives of this investigation are as follows: (1) to explore the volumetric and morphological alterations in the cervical longus and cervical extensor muscles subsequent to ACDF surgery and elucidate the underlying factors; (2) to analyze the potential correlation between changes in the cervical longus and cervical extensor muscles and the clinical efficacy observed following ACDF surgery.

This clinical retrospective study, conducted at a single center, adhered to the principles outlined in the Declaration of Helsinki. The research methodology was in accordance with the STROCSS criteria. Ethical approval for all procedures in this study was granted by the Ethics Committee of the Affiliated Hospital of Qingdao University. Prior to participation, written informed consent was obtained from all individuals involved in the study.

### Inclusion criteria

The inclusion criteria were as follows: (1) patients diagnosed with CSM for whom conservative treatments proved ineffective, necessitating surgical intervention; (2) individuals subjected to single-segment ACDF surgery, employing cervical interbody fusion cage and titanium plate, with all surgical procedures conducted by a consistent surgeon; (3) primary outcome measures encompassed the assessment of volume and morphological changes in the cervical longus and cervical extensor muscles, alongside the evaluation of clinical efficacy; and (4) the study incorporated imaging measurements as a methodological component.

### Exclusion criteria

Exclusion criteria were as follows: (1) patients presenting cervical vertebra trauma, infection, or tumor; (2) individuals with rheumatoid arthritis or ankylosing spondylitis; (3) patients displaying congenital cervical deformity; (4) individuals with a history of prior cervical vertebra surgery; and (5) those with incomplete imaging data, including anteroposterior and lateral radiographs, as well as MRI scans of the cervical spine, both preoperatively and throughout the follow-up period.

### Research method

#### Measurement of imaging parameters

The MRI examination was conducted utilizing a 1.5 T magnetic resonance scanner manufactured by Siemens Medical Solutions, Germany. The scanning protocol encompassed the region from the occiput to T2, encompassing the entirety of cervical flexor and extensor tissues. The acquired MRI scanning images featured a slice thickness of 4 mm, with an inter-image interval of 0.4 mm. T2-weighted imaging (T2WI) sequences, aligned with the upper endplate, equatorial line, and lower endplate of the intervertebral disk, were obtained for the following cervical segments: C2–3, C3–4, C4–5, C5–6, and C6–7 [11]. Within this study, patient imaging data were retrieved through the Picture Archiving and Communication System (PACS) of our hospital. Two medical professionals utilized Image Pro Plus 6.0 software (MEDIA CYBERNETICS, USA) independently to manually delineate the boundaries of neck muscles on each MRI scan. The assessed musculature comprises the cervical longus and principal extensor muscles, encompassing multifidus, hemispinalis cervicis, hemispinalis capitis, splenius capitis, and splenius colli. In each segment, the average value derived from measurements at three distinct levels was calculated, and the final average value obtained by two image measurers was utilized for subsequent statistical analysis.

#### Consistency check

Imaging parameters were independently measured by two spine surgeons. The results obtained by the two measurers underwent scrutiny to ensure inter-rater consistency.

### Observation target

#### Axial section of longus colli cross-sectional area (AxCSA) and cervical extensor cross-sectional area (CESA)

**Fig. 1:**
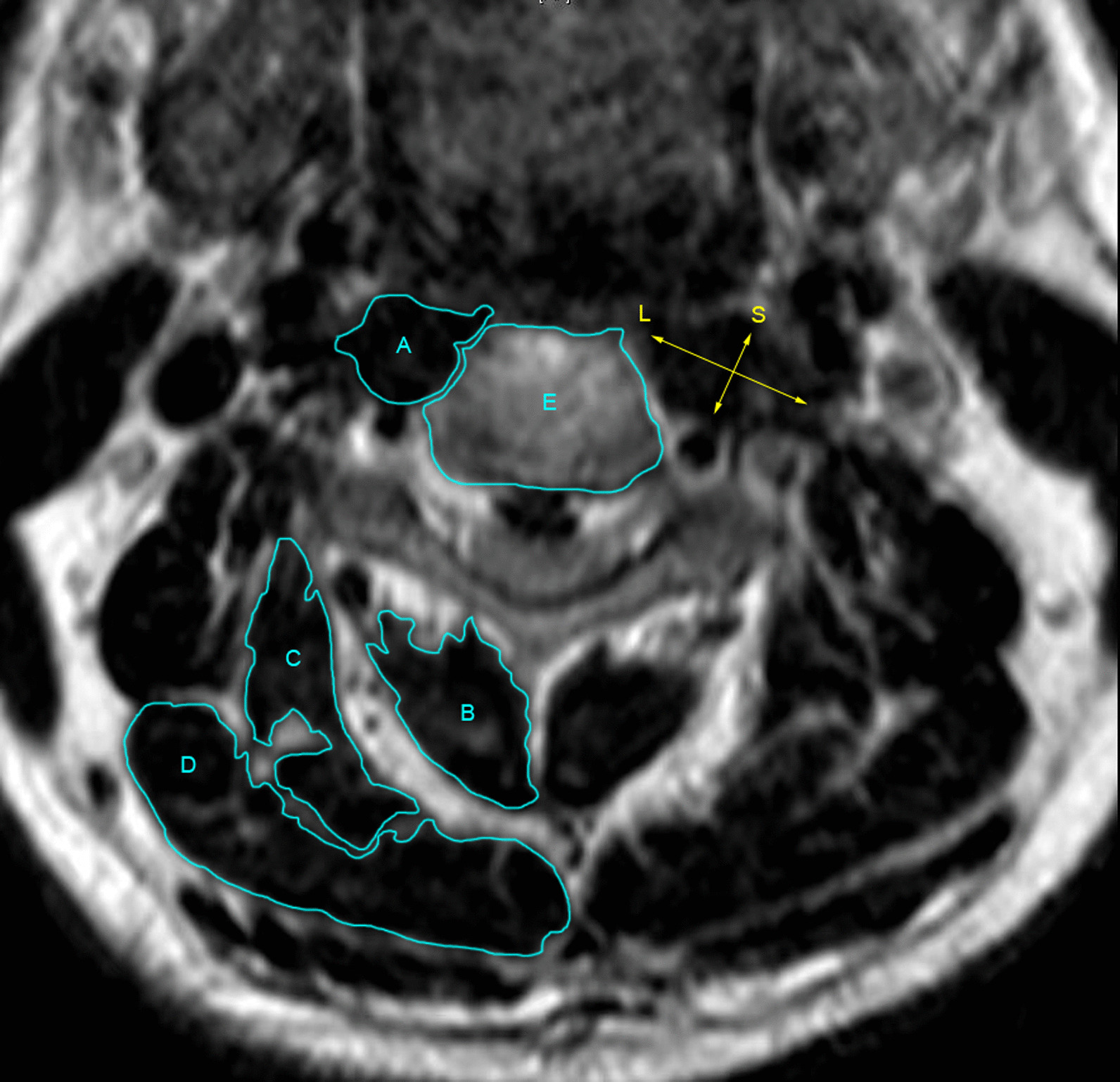
Measurement diagram of cervical longus, cervical extensor muscles, vertebral body area, and ratio of long and short diameter line (RLS) of cervical longus in axial position. A. cervical longus; B. multifidus muscle + semispinalis cervicalis muscle; C. splenius cervicis muscle + musculus semispinalis capitis; D. splenius capitis. The long (L) and short (S) lines must be intersected in the closed figure of the cross section and perform “dichotomy cutting” on the closed figure. RLS = L/S

The cross-sectional area of muscle tissue at an axial position is characterized as the aggregate of visually measured areas within the boundary of muscular fascial tissue on the T2WI of cervical spine MRI, aligning parallel to the intervertebral disk level (Fig. 1). Simultaneously, the cross-sectional area of the corresponding vertebral body was measured and designated as the vertebral body area (VBA).

#### Ratio of long and short lines (RLS)

The RLS is calculated as the quotient of the longest diameter to the shortest diameter observed in the cross-sectional view of the cervical longus. This metric serves as a vivid indicator of the morphological alterations in the cervical longus. It is imperative that the long and short lines intersect within the closed figure of the cross section, thereby facilitating a “dichotomy cutting” of the enclosed shape (Fig. 1).

#### Quantification of cervical longus volume and cervical extensor volume

To quantify the muscle volume of the cervical longus, the boundaries of the neck muscle were manually delineated on each MRI image (Fig. 1). The integration algorithm was subsequently employed to calculate the muscle volume based on the cross-sectional area of the muscle and the slice thickness. The calculated muscle volume from all MRI images was then aggregated to determine the overall volume of the cervical longus. The quantification of cervical extensor volume followed the measurement method proposed by Kim et al. [12], specifically utilizing the ratio of CESA to VBA, thereby mitigating potential measurement errors arising from variations in patient body size.

#### Visual analog scale (VAS)

The VAS score serves as a subjective method for evaluating pain intensity, as reported by the patient. In this scoring system, zero and ten, respectively, denote the absence of pain or the maximum perceived pain, and the patient selects one of the eleven numbers to articulate the intensity of pain according to their personal experience. The scoring scale ranges from 0 to 10 points, with pain intensity categorized as follows: 2–4 points for mild pain, 5–7 points for moderate pain, and 8–9 points for severe pain. The pain intensity experienced by the patient in the neck and shoulder was assessed preoperatively, at three and twelve months postoperatively, and during the final follow-up.

#### Modified Japanese Orthopedic Association score (mJOA)

The mJOA score is an assessment method developed by the Japanese Orthopedic Society specifically designed for evaluating nerve function in the cervical spinal cord. The total score on this scale is eighteen points, encompassing motor dysfunction scores for the upper extremities (5 points), motor dysfunction scores for the lower extremities (7 points), sensation (3 points), and sphincter dysfunction (3 points). A decline in the score indicates a progressive exacerbation of spinal cord dysfunction. The mJOA score for patients with CSM can be stratified into categories of mild myelopathy (mJOA from 15 to 17), moderate myelopathy (mJOA from 12 to 14), and severe myelopathy (mJOA from 0 to 11).

#### Neck disability index (NDI)

The NDI questionnaire comprises 10 items, encompassing aspects such as pain intensity, personal care, lifting, reading, headaches, concentration, work, driving, sleeping, and recreation. Each item is scored on a scale from 0 to 5 points. The NDI serves as a tool to gauge the extent of the impact of cervical disease on the daily life of the respondent, with a higher total score indicating a greater level of disability. The NDI percentage is calculated using the formula: NDI (%) = (total score/number of items completed by the subject × 5) × 100%.

#### Clinical efficacy

The clinical efficacy was assessed using the Odom criteria during the final follow-up. Succinctly, the outcomes were classified into four categories: excellent, good, satisfactory, and poor. An “excellent” rating indicates the absence of symptoms related to cervical disease, allowing the patient to engage in daily activities without limitations. A “good” rating denotes moderate symptoms related to cervical disease with the ability to perform daily activities without significant constraints. “Satisfactory” signifies a slight improvement in cervical disease symptoms but notable limitations in daily activities. Lastly, a “poor” rating suggests no improvement or worsening of cervical disease symptoms, rendering the patient unable to perform daily activities.

### Statistical analysis

Statistical analyses and graphical representations were conducted using SPSS 22.0 (SPSS, USA) and GraphPad 8.0.2 (GraphPad Software, USA), respectively. The intraclass correlation coefficient (ICC) test was employed to assess the consistency of measurement data obtained by two measurers. A consistency level of ICC ≥ 0.75 was considered good, while ICC values between 0.40 and 0.75 denoted average consistency, and ICC ≤ 0.40 indicated poor consistency. Analysis of variance was employed to compare the differences in AxCSA, RLS, CESA, and CESA/VBA at various preoperative and postoperative follow-up time points for patients. Paired t tests were selected for AxCSA, RLS, CESA, and CESA/VBA to compare preoperative data with data at each postoperative time point. Pearson correlation coefficient analysis was used to explore the correlation between AxCSA, RLS, CESA, and CESA/VBA, and mJOA, VAS, and NDI at the 3rd, 12th month, and the final follow-up after surgery. A two-sided alpha level of 0.05 was adopted for significance testing in this study.

## Results

Between January 2013 and December 2022, a total of 753 cases of CSM patients underwent single-segment ACDF surgery at the Spine Surgery Department of the Affiliated Hospital of Qingdao University. After applying the specified inclusion and exclusion criteria, our study focused on 65 patients (38 males, 27 females). The average age of these patients was 52.23 ± 10.8 years, ranging from 27 to 73 years. The surgical segments and corresponding case distribution were as follows: C2–3 segment (10 cases), C3–4 segment (12 cases), C4–5 segment (16 cases), C5–6 segment (14 cases), and C6–7 segment (13 cases). All patients presented symptoms of cervical spinal cord compression, including neck and shoulder discomfort, as well as numbness in one or both upper limbs, exacerbated by activity and relieved by rest. Additionally, 26 cases (40%) exhibited walking instability characterized by unsteady gait and a sensation of walking on cotton. Anteroposterior and lateral radiographs of the cervical spine revealed a reduction in the height of the intervertebral space at the affected segment. The CT and MRI scans indicated cervical disk herniation, thickening of the posterior longitudinal ligament, and ossification of the posterior longitudinal ligament. Notably, 20 patients (30.77%) exhibited signs of spinal cord degeneration.

### Clinical efficacy

In the final follow-up assessment of the 65 patients, 40 cases (61.54%) were categorized as excellent, 23 cases (35.38%) as good, and 2 cases (3.08%) as average, based on the Odom criteria.

### Consistency test results

Due to the extensive volume of measurement data, illustrative examples were extracted from the preoperative measurements of AxCSA, RLS, and CESA at the C2-3 level. The ICC values for AxCSA, RLS, and CESA, between the two measurers, were 0.976 (95% CI 0.962, 0.986), 0.993 (95% CI 0.989, 0.996), and 0.975 (95% CI 0.960, 0.985), respectively. The outcomes of the consistency test demonstrated that all ICC values exceeded 0.75, signifying a high level of agreement between the measurements conducted by the two observers.

### Changes of AxCSA and CESA after surgery

There are significant statistical differences in the AxCSA of the operative segment at various preoperative and postoperative follow-up time points (*F* = 832.2, *P* < 0.0001). The AxCSA of the operative segment exhibited a consistent reduction in postoperative measurements at the 3rd month, 12th month, and the last follow-up compared to preoperative values (141.62 ± 19.78 mm^2^). The postoperative AxCSA data at these respective time points were (119.42 ± 20.08) mm^2^ (*P* < 0.001), (117.59 ± 19.69) mm^2^ (*P* < 0.001), and (117.41 ± 19.19) mm^2^ (*P* < 0.001), indicating statistically significant differences. The postoperative AxCSA measurements at these intervals reflected a decrease of 15.68%, 16.97%, and 17.10% in comparison with preoperative data. Conversely, for the AxCSA of the non-operative segment, no significant differences were observed between the preoperative data and the postoperative data obtained at each follow-up time point (*P* > 0.05). Additionally, there were no significant differences in the AxCSA for the non-operative segment between preoperative and postoperative follow-up time points (*F* = 0.8159, *P* = 0.4641).

**Fig. 2:**
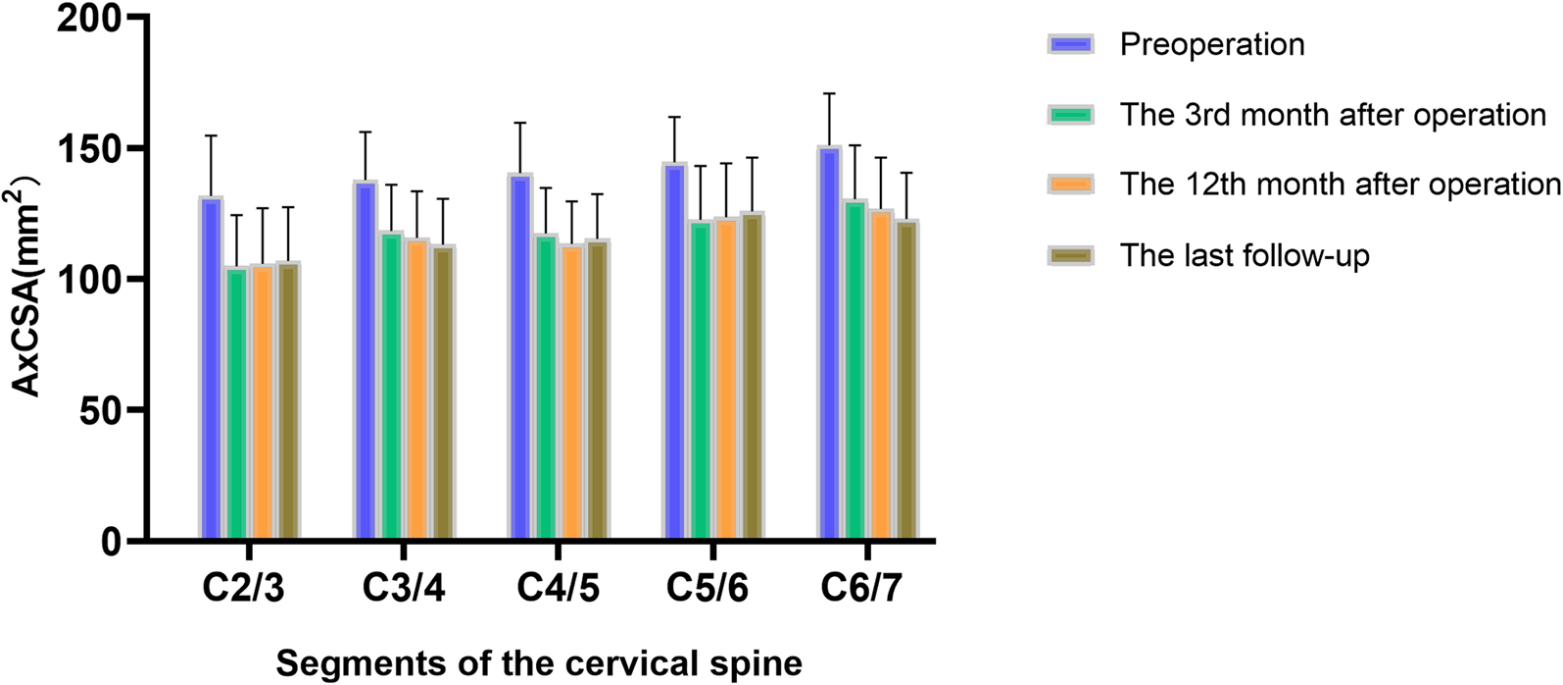
Schematic diagram of the changes in the cross-sectional area of the cervical longus (AxCSA) at the surgical segment before and after surgery. The data show a significant decrease in AxCSA at each surgical segment after surgery, which indicates that the cervical longus underwent a certain extent of atrophy after ACDF surgery

In the context of the AxCSA analysis pertaining to the C2-3 vertebral segment, the measurement results at various preoperative and postoperative follow-up time points showed significant statistical differences (*F* = 264.3, *P* < 0.0001). The preoperative data exhibited a mean value of (131.81 ± 22.88) mm^2^, subsequent to the surgical intervention, a discernible reduction was observed in the postoperative measurements, with values recorded as (104.88 ± 19.57) mm^2^ (*P* < 0.001), (105.86 ± 21.27) mm^2^ (*P* < 0.001), and (107.01 ± 20.45) mm^2^ (*P* < 0.001) for the respective analyses, as illustrated in Fig. 2. The observed disparities between preoperative and postoperative AxCSA measurements were determined to be statistically significant (Fig. 2).

In the context of the AxCSA analysis related to the C3–4 vertebral segment, the measurement results at various preoperative and postoperative follow-up time points showed significant statistical differences (*F* = 186.4, *P* < 0.0001). The preoperative data demonstrated a mean value of (137.86 ± 18.21) mm^2^, following the surgical intervention, a noteworthy reduction in postoperative measurements was observed, registering as (118.39 ± 17.64) mm^2^ (*P* < 0.001), (115.80 ± 17.71) mm^2^ (*P* < 0.001), and (113.13 ± 17.58) mm^2^ (*P* < 0.001) for the respective analyses, as delineated in Fig. 2. These observed discrepancies between preoperative and postoperative AxCSA measurements were found to be statistically significant (Fig. 2).

In the examination of AxCSA within the C4–5 vertebral segment, the measurement results at various preoperative and postoperative follow-up time points showed significant statistical differences (*F* = 482.3, *P* < 0.0001). The preoperative data revealed a mean value of (140.41 ± 19.24) mm^2^, following the surgical intervention, a discernible reduction in postoperative measurements was evident, registering at (117.40 ± 17.32) mm^2^ (*P* < 0.001), (113.42 ± 16.23) mm^2^ (*P* < 0.001), and (115.31 ± 17.16) mm^2^ (*P* < 0.001) for the respective assessments, as depicted in Fig. 2. These variations between preoperative and postoperative AxCSA values were determined to be statistically significant, as illustrated in Fig. 2.

In the context of the AxCSA analysis pertaining to the C5–6 spinal segment, the measurement results at various preoperative and postoperative follow-up time points showed significant statistical differences (*F* = 194.7, *P* < 0.0001). The preoperative measurements revealed a mean value of (144.50 ± 17.46) mm^2^, following the surgical intervention, the corresponding postoperative measurements exhibited a discernible reduction to (122.62 ± 20.56) mm^2^ (*P* < 0.001), (123.66 ± 20.46) mm^2^ (*P* < 0.001), and (125.81 ± 20.51) mm^2^ (*P* < 0.001), as illustrated in Fig. 2. These alterations in postoperative measurements achieved statistical significance, as denoted in Fig. 2.

In the analysis of the AxCSA pertaining to the C6–7 segment, the measurement results at various preoperative and postoperative follow-up time points showed significant statistical differences (*F* = 244.0, *P* < 0.0001). The preoperative measurement yielded (151.00 ± 19.88) mm^2^, subsequent to the surgical intervention, the postoperative assessments revealed a notable reduction to (130.56 ± 20.42) mm^2^ (*P* < 0.001), (126.84 ± 19.52) mm^2^ (*P* < 0.001), and (122.91 ± 17.70) mm^2^ (*P* < 0.001), as depicted in Fig. 2. Importantly, these postoperative alterations in AxCSA values were found to be statistically significant, as underscored in Fig. 2.

In the analysis of CESA within both operative and non-operative segments, no statistically significant distinctions were observed when comparing preoperative data with postoperative data at the 3rd month, 12th month, and the final follow-up (*P* > 0.05). Meanwhile, there were no significant differences in the CESA between preoperative and postoperative follow-up time points for the operative segment (*F* = 2.506, *P* = 0.1157), and no significant differences were observed in CESA for the non-operative segment between preoperative and postoperative follow-up time points (*F* = 1.003, *P* = 0.3567).

### Postoperative changes of RLS

There are significant statistical differences in the RLS of the operative segment at various preoperative and postoperative follow-up time points (*F* = 513.4, *P* < 0.0001). In the context of RLS assessment within the operative segment, a discernible elevation was noted in comparison with the preoperative baseline value of 2.05 ± 0.57. Postoperative data at the 3rd month, 12th month, and the final follow-up displayed increments to 2.80 ± 0.51 (*P* < 0.001), 2.84 ± 0.52 (*P* < 0.001), and 2.83 ± 0.53 (*P* < 0.001), respectively, with each difference being statistically significant. These increments corresponded to rates of increase amounting to 36.59%, 38.54%, and 38.05%, sequentially. Conversely, for the non-operative segment’s RLS, no statistically significant differences were identified when comparing preoperative and postoperative data at each follow-up time point (*P* > 0.05). Furthermore, there were no significant differences in the RLS for the non-operative segment between preoperative and postoperative follow-up time points (*F* = 3.502, *P* = 0.0515).

**Fig. 3:**
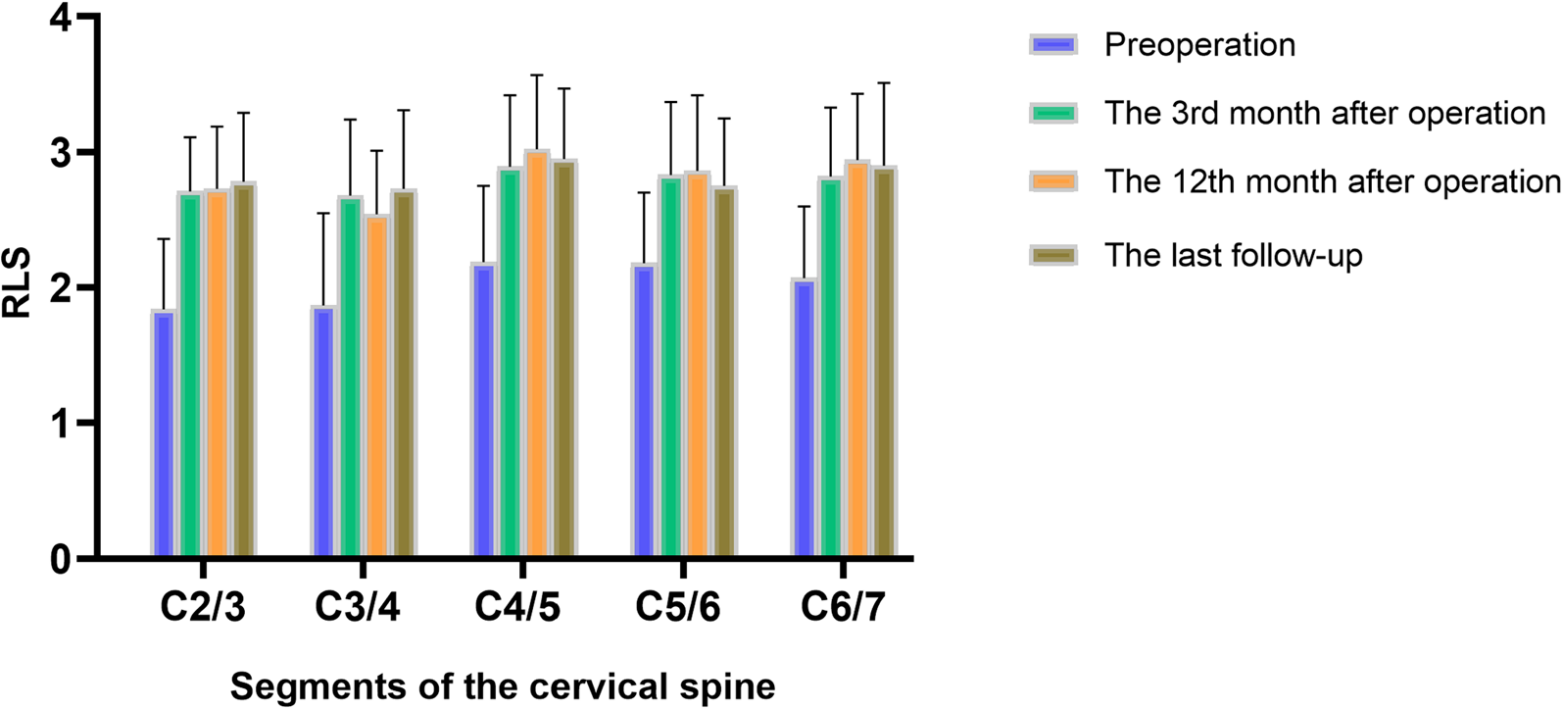
Schematic diagram of changes in the ratio of long and short diameter line (RLS) of the cervical longus at the surgical segment before and after surgery. The postoperative RLS of each surgical segment increased, which indicates that the cross-sectional morphology of the cervical longus changed from “circular” to “elliptical”

Statistically significant differences in RLS were observed within the C2–3 segment (*F* = 269.3, *P* < 0.0001). The preoperative RLS was recorded as 1.84 ± 0.52, and subsequent to the surgical intervention, postoperative values demonstrated a notable increase to 2.71 ± 0.40 (*P* < 0.001), 2.73 ± 0.46 (*P* < 0.001), and 2.78 ± 0.51 (*P* < 0.001), as illustrated in Fig. 3.

Statistically significant disparities in RLS were evident within the C3–4 segment (*F* = 30.93, *P* < 0.0001). The preoperative RLS registered at 1.87 ± 0.68, while postoperative assessments exhibited a notable escalation to 2.68 ± 0.56 (*P* < 0.001), 2.54 ± 0.47 (*P* = 0.001), and 2.73 ± 0.58 (*P* < 0.001), as delineated in Fig. 3.

Statistically significant discrepancies in RLS were identified within the C4–5 segment (*F* = 909.8, *P* < 0.0001). The preoperative RLS, quantified at 2.19 ± 0.56, witnessed a notable increase in the postoperative period to 2.89 ± 0.53 (*P* < 0.001), 3.02 ± 0.55 (*P* < 0.001), and 2.95 ± 0.52 (*P* < 0.001), as depicted in Fig. 3.

Significant statistical disparities in RLS were discerned within the C5–6 segment (*F* = 345.4, *P* < 0.0001). The initial preoperative RLS measurement, recorded at 2.18 ± 0.52, exhibited a substantial postoperative augmentation to 2.83 ± 0.54 (*P* < 0.001), 2.86 ± 0.56 (*P* < 0.001), and 2.75 ± 0.50 (*P* < 0.001), as illustrated in Fig. 3.

Statistically significant disparities in RLS were evident within the C6–7 segment (*F* = 329.5, *P* < 0.0001). The preoperative RLS measurement, initially quantified at 2.07 ± 0.53, demonstrated a notable postoperative increase to 2.82 ± 0.51 (*P* < 0.001), 2.94 ± 0.49 (*P* < 0.001), and 2.90 ± 0.61 (*P* < 0.001), as depicted in Fig. 3.

### Postoperative changes of the volume of cervical longus and CESA/VBA

**Fig. 4:**
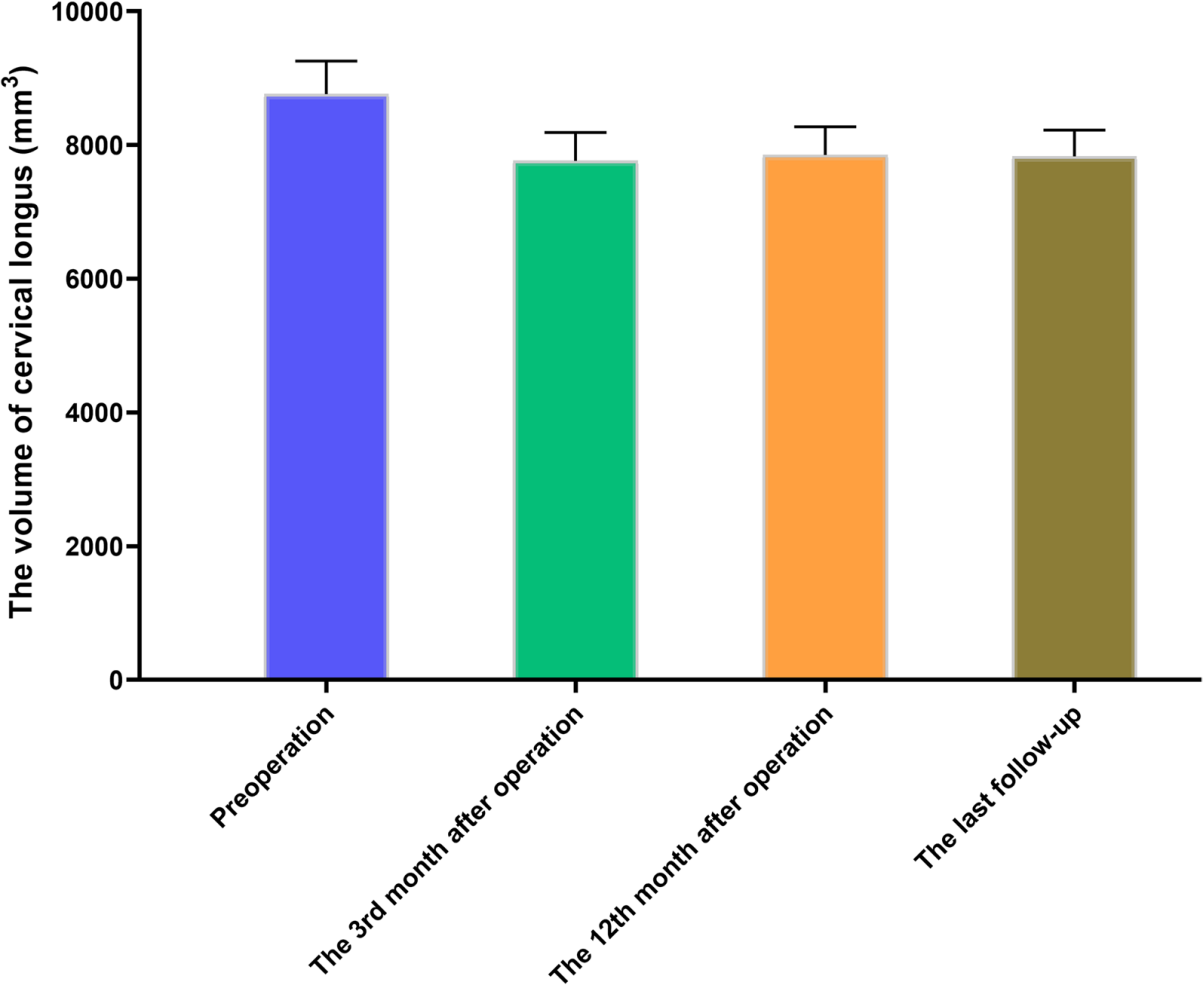
Schematic diagram of changes in the volume of the cervical longus after surgery. The volume of the cervical longus decreased significantly at each follow-up time point after surgery, which indicates a certain degree of atrophy of the cervical longus

There were significant differences in the volume of the cervical longus between preoperative, 3 months postoperative, 12 months postoperative, and the last follow-up (*F* = 4109, *P* < 0.0001). Meanwhile, discernible alterations were observed when comparing postoperative data at the 3rd month, 12th month, and last follow-up to the preoperative baseline (8764.69 ± 492.91 mm^3^). Statistically significant reductions were noted in the postoperative measurements, amounting to (7762.16 ± 427.98) mm^3^ (*P* < 0.001), (7852.68 ± 422.06) mm^3^ (*P* < 0.001), and (7830.45 ± 394.44) mm^3^ (*P* < 0.001), respectively. The corresponding rates of decrease were calculated at 11.44%, 10.41%, and 10.66%, as illustrated in Fig. 4.

**Fig. 5:**
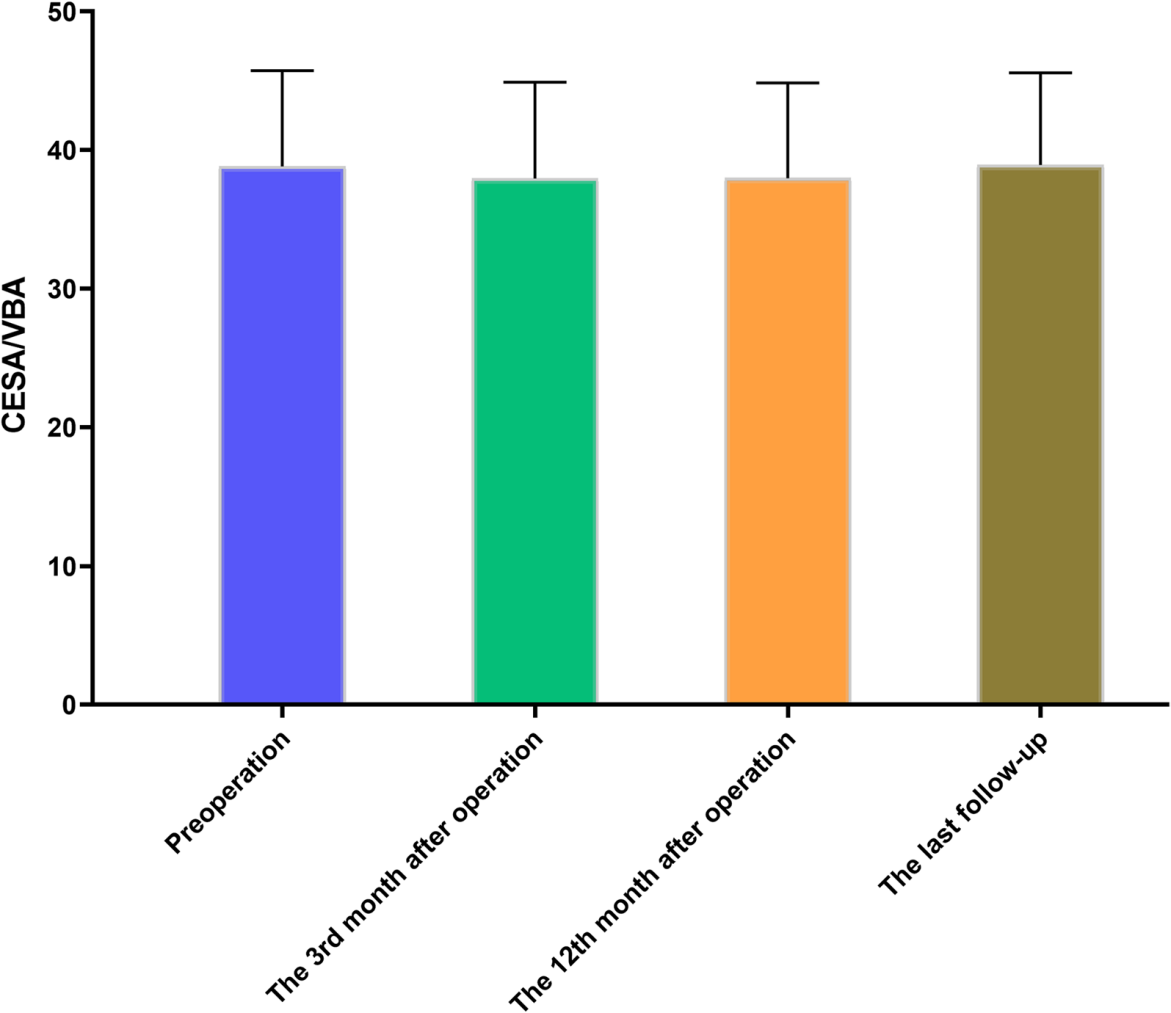
Schematic diagram of changes in the volume of cervical extensor muscles (CESA/VBA) after surgery. No statistically significant differences were identified between the preoperative CESA/VBA and the corresponding postoperative values at each follow-up time point (*P* > 0.05). This suggests that anterior cervical surgery may not exert a substantial impact on the cervical extensor muscles

In the evaluation of CESA/VBA, no statistically significant differences were observed between the preoperative data and postoperative measurements at each follow-up time point (*P* > 0.05), as depicted in Fig. 5. However, there were significant differences in the CESA/VBA between preoperative and postoperative follow-up time points (*F* = 5.733, *P* = 0.0123).

### VAS

In the evaluation of the VAS, discernible changes were noted in comparison with the preoperative baseline (3.11 ± 1.06 points). The postoperative data at the 3rd month, 12th month, and last follow-up revealed significant decreases, registering at (1.46 ± 0.92) points (*P* < 0.001), (1.06 ± 0.93) points (*P* < 0.001), and (0.82 ± 0.85) points (*P* < 0.001), respectively.

### NDI

In the assessment of the NDI, discernible changes were observed in contrast to the preoperative baseline (16% ± 4%). The postoperative data at the 3rd month, 12th month, and last follow-up indicated statistically significant reductions, recording values of (12% ± 5%) (*P* < 0.001), (6% ± 4%) (*P* < 0.001), and (7% ± 2%) (*P* < 0.001), respectively.

### mJOA

In relation to the mJOA scores, distinct changes were observed in comparison with the preoperative baseline (13.71 ± 1.60 points). Postoperative assessments at the 3rd month, 12th month, and last follow-up revealed statistically significant increases, registering at (15.38 ± 1.09) points (*P* < 0.001), (15.85 ± 0.97) points (*P* < 0.001), and (15.98 ± 0.82) points (*P* < 0.001), respectively.

### Correlation analyses between the volume of cervical longus or CESA/VBA and postoperative scores (VAS, mJOA, and NDI)

**Fig. 6:**
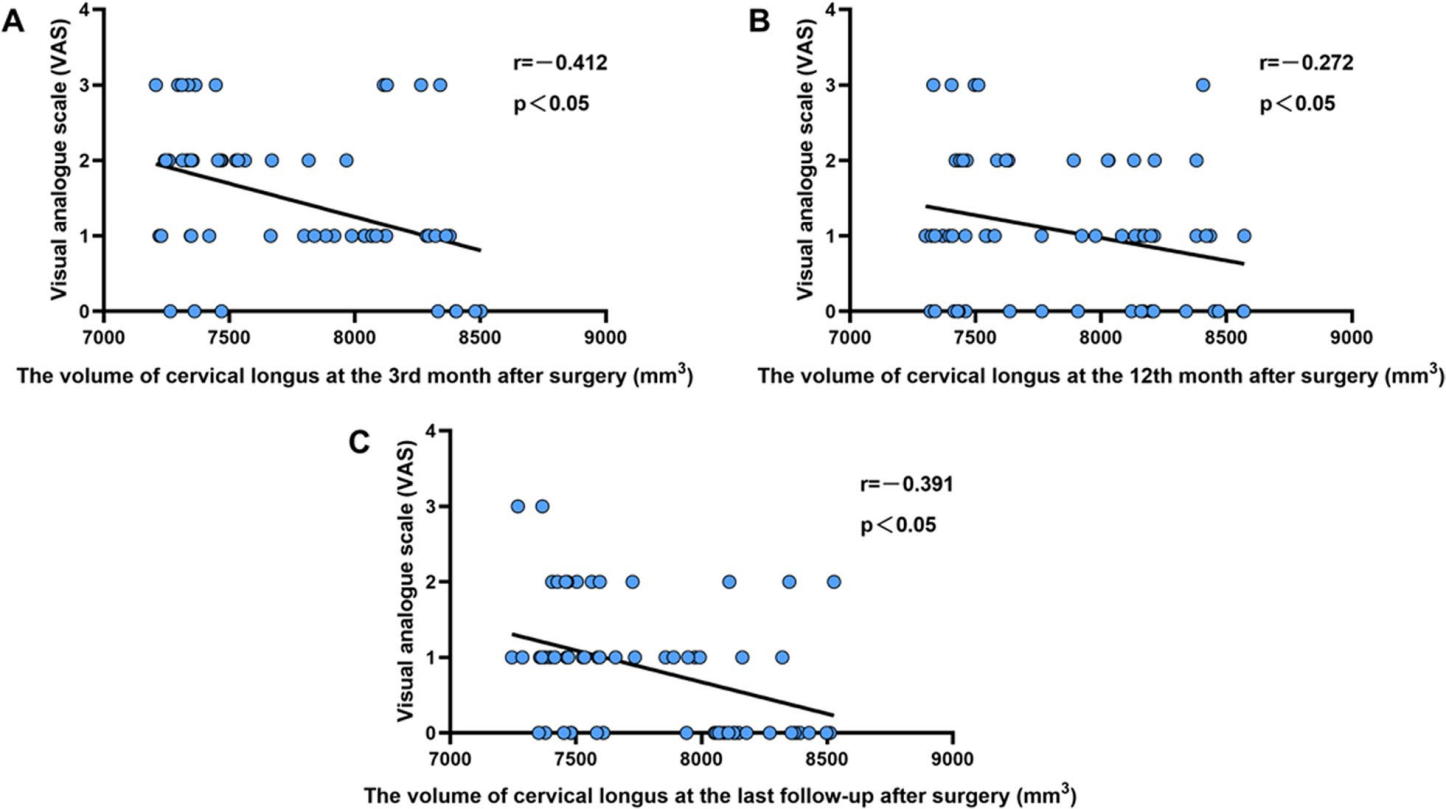
Analysis of the correlation between the volume of cervical longus and VAS score in 65 patients at the 3rd month (**A**), 12th month (**B**), and the last follow-up (**C**) after surgery. There was a negative correlation between the volume of cervical longus and VAS score, which indicates that the decrease in the volume of cervical longus may be the cause of postoperative neck pain

**Fig. 7:**
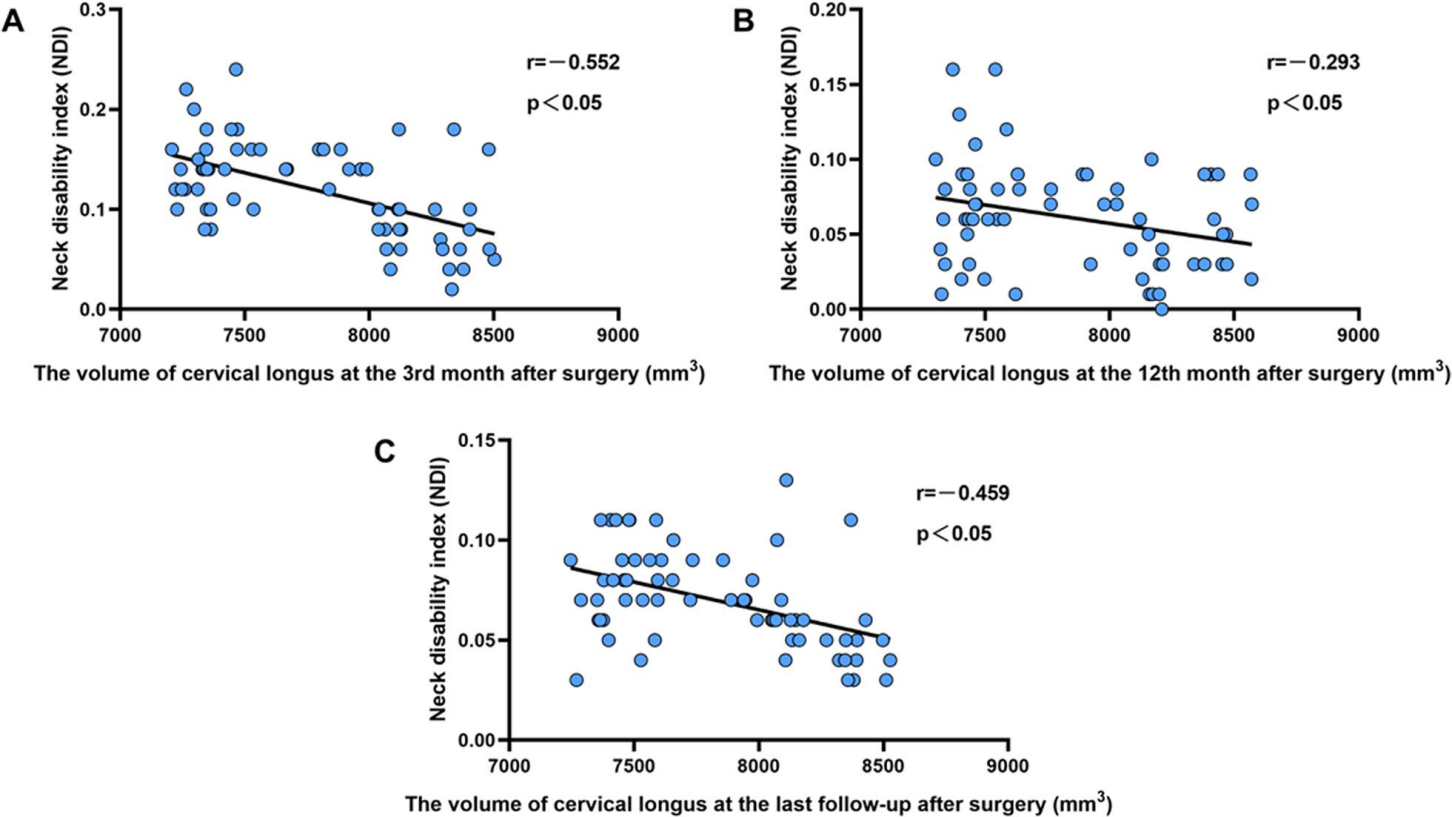
Analysis of the correlation between the volume of cervical longus and NDI in 65 patients at the 3rd month (**A**), 12th month (**B**), and the last follow-up (**C**) after surgery. There was a negative correlation between the volume of cervical longus and NDI, which indicates that the decrease in the volume of cervical longus may be the cause of postoperative neck pain

Correlation analyses were separately conducted between the volume of the cervical longus or CESA/VBA and postoperative scores (VAS, mJOA, and NDI). The results revealed negative correlations between the volume of cervical longus and VAS at the 3rd (*r* = − 0.412, *P* < 0.001), 12th (*r* = − 0.272, *P* = 0.0287) month, and the last follow-up (*r* = − 0.391, *P* = 0.0013) (Fig. 6). Negative correlations were also observed between the volume of cervical longus and NDI at the 3rd (*r* = − 0.552, *P* < 0.001), 12th (*r* = − 0.293, *P* = 0.0178) month, and the last follow-up (*r* = − 0.459, *P* < 0.001) (Fig. 7). However, no significant correlation was identified between the volume of cervical longus and mJOA (*P* > 0.05). Furthermore, no correlation was found between CESA/VBA and postoperative scores (VAS, mJOA, and NDI) (*P* > 0.05).

## Discussion

### The volumetric and morphological alterations in the cervical longus and cervical extensor muscles subsequent to ACDF surgery and the underlying causative factors

The present study discerned notable alterations in cervical longus among patients who underwent ACDF surgery, characterized by a reduced muscle volume, diminished AxCSA, and an enlarged RLS. Conversely, no significant changes were observed in the muscle volume of cervical extensors between the preoperative data and the postoperative data at each follow-up time point. However, there were significant differences in the muscle volume of cervical extensors between preoperative and postoperative follow-up time points. The reduction in the volume of cervical longus may be attributed to several potential causes. In the course of ACDF surgery, unavoidable soft tissue damage occurs in the anterior portion of the neck due to the application of surgical instruments. This damage encompasses stretching injuries to the muscle tissue, stimulation of the paravertebral muscles by surgical implants, and thermal damage resulting from the use of high-frequency electrotome [13]. The injury to the cervical longus and subsequent scar repair of muscle tissue following ACDF surgery may contribute to the observed reduction in the volume of the cervical longus and AxCSA of the operative segment to a certain extent. While the necessity of postoperative cervical collar use remains a subject of debate, it is customary for patients to don a cervical collar during the initial postoperative period. The primary objective is to limit neck movements, thereby aiming to enhance fusion rates [14]. The utilization of a cervical collar can elicit discernible alterations in the neck region, potentially leading to muscle atrophy. Individuals exhibiting heightened apprehension of pain may actively abstain from engaging in physical activities anticipated to induce or exacerbate pain—a phenomenon recognized as fear-avoidance beliefs [15]. Fear-avoidance behavior can potentially engender deficiencies in the postoperative rehabilitation exercise and hinder the functional recovery of affected patients. This, in turn, may contribute to a reduction in the volume of the cervical longus. Additionally, the fusion of cervical segments may result in a diminished range of motion, thereby influencing the volumetric changes observed in cervical muscles. Consequently, alterations in the cervical longus may be attributed to a combination of muscle disuse and inhibition [8, 16]. Intervertebral disk degeneration and its associated inflammatory mediators emerge as pivotal factors influencing the structural alterations observed in paravertebral muscles [17, 18]. A prior animal study has demonstrated that intervertebral disk degeneration, coupled with associated inflammatory changes, can induce atrophy of the Lumbar Multifidus [19]. Diverse inflammatory mediators can intricately contribute to the promotion of muscle fat infiltration and fibrosis, ultimately culminating in muscle atrophy and degeneration [17, 19]. The findings from animal studies align fundamentally with the observations of muscle atrophy and fat infiltration in human studies [20–22]. The findings of this study indicate that, compared with the preoperative data, the postoperative cross-sectional area and muscle volume of cervical extensors showed no significant changes, while CESA/VBA experienced a minor reduction at the 3rd and 12th months post-operation, followed by a slight increase at the final follow-up. There is a significant intergroup difference in the measurements of CESA/VBA between the preoperative and postoperative assessments. ACDF serves the purpose of reconstructing cervical vertebral stability; however, the fusion of cervical vertebrae in the operative segments results in the loss of their original mobility. Consequently, an augmented mechanical stress in adjacent cervical segments ensues, potentially contributing to an accelerated degeneration of intervertebral disks in these neighboring segments [23, 24]. The aforementioned accelerated degeneration of the cervical intervertebral disk could be a contributing factor to the observed declining trend in the muscle volume of cervical extensors. Additionally, the reduction in neck movement caused by factors such as postoperative wearing of cervical collar may lead to atrophy of the cervical extensors. Subsequent factors, such as increased neck activity and rehabilitation exercises, may contribute to an increase in the volume of cervical extensors. The postoperative increase in RLS suggests a transformation in the cross-sectional shape of the cervical longus from a “round” configuration to an “oval” configuration following ACDF. A prior investigation has substantiated that ACDF possesses the capability to rectify the curvature of the cervical spine, thereby augmenting cervical lordosis, particularly in patients exhibiting sagittal imbalance of the cervical spine. Furthermore, the extent of correction in cervical curvature post-surgery is more pronounced in cases where the cervical curvature is initially smaller [25]. Such alteration in cervical curvature exerts a specific elongating influence on the cervical longus. Under these circumstances, both volume and muscle bundle reconstruction of the cervical longus occur, as there is no discernible change in the sarcolemma. Consequently, the cervical longus exhibits a diminished thickness compared to its preoperative state. Additionally, a previous investigation into degenerative cervical spine disease revealed that individuals with chronic neck pain presented a broader ovoid shape of the cervical multifidus when compared to their healthy counterparts [6]. Hence, cervical pain could be regarded as a potential influencing factor capable of altering the morphology of deep cervical muscles. Additionally, various factors may influence the neck muscles post-ACDF, including patient age, gender, the cervical spine segment of the surgery, and the duration of the operation. This study conducted a detailed investigation into the changes in the cervical longus and cervical extensor muscles post-ACDF across different cervical spine segments. Regarding the impact of age, gender, and operation duration on post-ACDF neck muscles, relevant clinical studies indicate: (1) The influence of gender does not seem to exert a substantial impact on the clinically meaningful recovery subsequent to single-level ACDF [26]; (2) a study found that patients’ age is one of the factors affecting the effectiveness of ACDF surgery. Younger patients achieved better surgical outcomes in terms of cervical lordosis correction and graft height loss after ACDF. The correction of cervical lordosis and loss of graft height are closely related to changes in cervical muscles. Therefore, this result also indicates that patient age is one of the factors influencing post-ACDF changes in cervical muscles [27]; (3) a study revealed that prolonging the duration of single-level ACDF surgery is associated with an increased incidence of postoperative swallowing difficulties. However, the observed differences did not reach statistical significance, possibly attributed to the limited number of cases. The manifestation of postoperative swallowing difficulties, to a certain extent, reflects the severity of damage to cervical muscles. Thus, the duration of surgery may indeed exert an impact on cervical muscle function [28].

### Correlation analysis of changes in volume of cervical longus and cervical extensor muscles with postoperative clinical efficacy and its significance

In the present study, noteworthy enhancements were observed in VAS, mJOA, and NDI scores postoperatively. These improvements signify the effectiveness of ACDF surgery in alleviating clinical symptoms in individuals with CSM. Nonetheless, it is noteworthy that a subset of postoperative patients continues to experience pain or other discomfort in the neck and shoulder region. In a cross-sectional magnetic resonance imaging study on idiopathic neck pain and cervical muscle volume, the findings indicated that cervical muscle volume serves as a contributing factor to the onset of idiopathic neck pain [29]. An analogous outcome has been reported in a study pertaining to lower back pain [30]. The findings of the current study revealed a negative correlation between the volume of the cervical longus and NDI as well as VAS, while no correlation was observed with mJOA. Conversely, there was no discernible correlation between the volume of cervical extensors and NDI, VAS, or mJOA. These results imply that the diminished volume of the cervical longus may be one of the potential factors contributing to postoperative idiopathic neck pain in individuals undergoing ACDF surgery. An augmentation in muscle volume appears to have a mitigating effect on cervical pain to some extent. Furthermore, no correlation was observed between the volume alteration of the cervical longus and the recuperation of postoperative neurological function. Previous report has indicated that diminished activation of the deep cervical flexor muscles is linked to a smaller muscle size. Consequently, the size of the deep cervical flexor muscles is intricately connected to their functional role in stabilizing the cervical vertebra [31]. Therefore, it is reasonable to posit that a smaller muscle volume signifies a weaker cervical longus, potentially leading to the instability of the cervical spine. A prior study has established an association between weaker deep cervical flexor muscles and cervical dysfunction [31]. The aforementioned findings align with the outcomes of our research. Additionally, pertinent clinical studies have demonstrated that cranio-cervical flexion exercises can augment the cross-sectional area of the cervical longus and mitigate neck pain, further corroborating our study results [32, 33].

### Limitations of the study

Our study is subject to two limitations. Firstly, the measurement and computation of AxCSA and RLS were manually conducted by clinicians using computer software. Several factors could potentially influence the accuracy of these measurements, including the patient’s posture during the magnetic resonance examination, artifacts from internal implants, soft tissue scar repair, and the anatomical relationship between the cervical longus and musculus longus capitis. To mitigate potential errors in the magnetic resonance image measurement and calculation process, we took measures to maintain patients in a standardized supine position during imaging examinations. Additionally, two experienced clinicians were enlisted to handle the imaging processing and data analysis. Furthermore, two radiologists were consulted to verify the delineation of the areas encompassing the cervical longus and cervical extensors on axial images. Secondly, fat infiltration of paraspinal muscles constitutes a prominent factor influencing muscle volume and function, correlating with postoperative functional recovery. However, this study does not delve further into elucidating the alterations and implications of muscle composition following ACDF surgery.

## Conclusion

This study suggests that ACDF may be a potential contributor to morphological changes in the cervical longus postoperatively, whereas ACDF exerts no significant impact on cervical extensors, including the multifid muscle, cervical semispinalis muscle, cephalic semispinalis muscle, splenius capitis, and splenius cervicis. Minimizing intraoperative cervical tissue disruption and enhancing postoperative rehabilitation training for cervical muscles could mitigate the postoperative reduction in cervical longus muscle volume, diminish the incidence of neck pain, and consequently enhance surgical outcomes while expediting postoperative recovery.

## Data Availability

The datasets used and/or analyzed during the current study are available from the corresponding author on reasonable request.

## Abbreviations

ACDF: Anterior cervical discectomy and fusion surgery
AxCSA: Axial section of longus colli cross-sectional area
RLS: Ratio of long and short diameter line
CESA: Cervical extensor cross-sectional area
VBA: Vertebral body area
VAS: Visual analog scale
mJOA: Modified Japanese Orthopedic Association score
NDI: Neck disability index
CSM: Cervical spondylotic myelopathy
ACDR: Artificial cervical disk replacement
T2WI: T2-weighted imaging
PACS: Picture archiving and communication system
ICC: Intraclass correlation coefficient
